# Strigolactones as an auxiliary hormonal defence mechanism against leafy gall syndrome in *Arabidopsis thaliana*

**DOI:** 10.1093/jxb/erv309

**Published:** 2015-06-30

**Authors:** Elisabeth Stes, Stephen Depuydt, Annick De Keyser, Cedrick Matthys, Kris Audenaert, Koichi Yoneyama, Stefaan Werbrouck, Sofie Goormachtig, Danny Vereecke

**Affiliations:** ^1^Department of Plant Systems Biology, VIB, 9052 Gent, Belgium; ^2^Department of Plant Biotechnology and Bioinformatics, Ghent University, 9052 Gent, Belgium; ^3^Department of Medical Protein Research, VIB, 9000 Gent, Belgium; ^4^Department of Biochemistry, Ghent University, 9000 Gent, Belgium; ^5^Ghent University Global Campus, Incheon 406-840, Republic of Korea; ^6^Department of Applied Biosciences, Ghent University, 9000 Gent, Belgium; ^7^Center for Bioscience Research & Education, Utsunomiya University, Utsunomiya 321–8505, Japan

**Keywords:** Apical dominance, Gram-positive phytopathogen, witches’ broom.

## Abstract

Leafy gall syndrome is the consequence of modified plant development in response to a mixture of cytokinins secreted by the biotrophic actinomycete *Rhodococcus fascians*. The similarity of the induced symptoms with the phenotype of plant mutants defective in strigolactone biosynthesis and signalling prompted an evaluation of the involvement of strigolactones in this pathology. All tested strigolactone-related *Arabidopsis thaliana* mutants were hypersensitive to *R. fascians*. Moreover, treatment with the synthetic strigolactone mixture GR24 and with the carotenoid cleavage dioxygenase inhibitor D2 illustrated that strigolactones acted as antagonistic compounds that restricted the morphogenic activity of *R. fascians*. Transcript profiling of the *MORE AXILLARY GROWTH1* (*MAX1*), *MAX2*, *MAX3*, *MAX4*, and *BRANCHED1* (*BRC1*) genes in the wild-type Columbia-0 accession and in different mutant backgrounds revealed that upregulation of strigolactone biosynthesis genes was triggered indirectly by the bacterial cytokinins via host-derived auxin and led to the activation of *BRC1* expression, inhibiting the outgrowth of the newly developing shoots, a typical hallmark of leafy gall syndrome. Taken together, these data support the emerging insight that balances are critical for optimal leafy gall development: the long-lasting biotrophic interaction is possible only because the host activates a set of countermeasures—including the strigolactone response—in reaction to bacterial cytokinins to constrain the activity of *R. fascians*.

## Highlight

To antagonize the developmental process initiated by *Rhodococcus fascians* and in response to the bacterial cytokinins, *Arabidopsis* activates its strigolactone response, partially suppressing shoot branching in the rosette.

## Introduction

Leafy gall syndrome is an infectious plant disease that affects a wide range of plants, primarily dicotyledonous herbs (for recent reviews, see Stes *et al.*, 2011*b*, 2013). The pathology is caused by the Gram-positive actinomycete *Rhodococcus fascians* and is characterized by the induction of multiple shoots, of which further outgrowth is inhibited. These shoots arise from the activation of dormant axillary meristems combined with the *de novo* formation of additional meristems (de O Manes *et al.*, 2001; Simón-Mateo *et al.*, 2006). The main virulence factor of *R. fascians* strain D188 is a cytokinin mix for which the biosynthetic machinery is encoded by the *fas* operon located on a linear virulence plasmid, pFiD188 (Pertry *et al.*, 2009, 2010; Francis *et al.*, 2012). The central gene of the operon, *fas*D, encodes an isopentenyltransferase that mediates the first dedicated step in cytokinin production (Pertry *et al.*, 2009, 2010). Although the plasmid-free derivative of strain D188, strain D188-5, also secretes low levels of cytokinins, they are insufficient to cause the disease (Pertry *et al.*, 2009, 2010). The perception of the bacterial cytokinins by plants is absolutely essential for leafy gall induction on all hosts because it stimulates cell proliferation, prevents tissue maturation, and converts infected regions into sink tissues (Depuydt *et al.*, 2008, 2009*a*, 2009*b*). Moreover, in *Arabidopsis*, the *fas* cytokinins also trigger increased production of other plant growth regulators, such as polyamines and auxins. More specifically, indole-3-acetic acid and putrescine have been shown to play accessory roles during symptom formation by activating meristem initiation and targeting expression of D3-type cyclins, respectively (Stes *et al.*, 2011*a*, 2012). In this manner, the relatively low amounts of cytokinins secreted by *R. fascians* can profoundly alter plant development. Nevertheless, additional hormones, such as abscisic acid and gibberellins, may be implicated in symptom development as well (Simón-Mateo *et al.*, 2006; Depuydt *et al.*, 2008), but their exact role in the pathology remains to be assessed. In *Arabidopsis*, *R. fascians* infection leads to delayed senescence, loss of apical dominance, activation of dormant axillary meristems, and formation of stunted inflorescences from dwarfed rosettes, altogether resulting in a bushy appearance (de O Manes *et al.*, 2004; Stes *et al.*, 2011*b*). Interestingly, these aspects of leafy gall syndrome resemble the phenotype of mutants impaired in strigolactone biosynthesis and/or sensing.

Strigolactones are apocarotenoids that have been classified as plant hormones. Together with auxins and cytokinins, they take a central position in the control of shoot branching (Gomez-Roldan *et al.*, 2008; Umehara *et al.*, 2008). Strigolactones have also been associated with other aspects of plant development (for recent reviews see Xie *et al.*, 2010; Brewer *et al.*, 2013; Waldie *et al.*, 2014). For instance, they control light-dependent photomorphogenesis during seed germination, influence root architecture, impact senescence, and affect flower development (Snowden *et al.*, 2005; Shen *et al.*, 2007; Agusti *et al.*, 2011; Domagalska and Leyser, 2011; Kapulnik *et al.*, 2011; Ruyter-Spira *et al.*, 2011; Rasmussen *et al.*, 2012; Shen *et al.*, 2012). Moreover, strigolactones appear to be linked to diverse abiotic stresses, such as nutrition (Bonneau *et al.*, 2013; Marzec *et al.*, 2013), drought, high salinity (Bu *et al.*, 2014; Ha *et al.*, 2014), and light stress (González-Pérez *et al.*, 2011; Jia *et al.*, 2014). However, the oldest known function of strigolactones is as rhizospheric host detection cues for root-parasitic plants and symbiotic arbuscular mycorrhizal fungi (Cook *et al.*, 1966; Akiyama *et al.*, 2005; Matusova *et al.*, 2005). More recently, strigolactones have also been implicated in other biotic interactions. For instance, strigolactones have been reported to affect nodule formation in diverse legumes upon interaction with their rhizobial partner (Soto *et al.*, 2010; Liu *et al.*, 2013; Foo *et al.*, 2014; De Cuyper *et al.*, 2015). In *Oryza sativa* (rice), the excess tillering observed after infection with rice grassy stunt virus has been associated with suppression of strigolactone biosynthesis and signalling genes (Satoh *et al.*, 2013). Finally, strigolactones might play a direct or indirect role in plant defence in different fungal pathosystems (Dor *et al.*, 2011; Torres-Vera *et al.*, 2013).

Strigolactones are derived from carotenoids and the first biosynthesis steps occur in the plastids. The β-carotene isomerase DWARF27 (D27) mediates the conversion of all-*trans*-β-carotene to 9-*cis*-β-carotene (Alder *et al.*, 2012), after which two carotenoid cleavage dioxygenases (CCDs), MORE AXILLARY GROWTH3 (MAX3) and MAX4 in *Arabidopsis*, cleave these intermediates to form carlactone. Carlactone moves from the plastids to the cytosol and undergoes oxidation by the cytosolic cytochrome P450 MAX1, resulting in the bioactive compounds (Booker *et al.*, 2005; Waters *et al.*, 2012; Abe *et al.*, 2014). Strigolactones are thought to be produced mainly in the roots and to be transported upwards in the xylem to inhibit bud outgrowth (Beveridge, 2006; Kohlen *et al.*, 2011). Two interacting proteins were shown to be central in strigolactone perception and signalling: D14/DAD2, an α/β-fold hydrolase proposed to be a strigolactone receptor (Waters *et al.*, 2012; Hamiaux *et al.*, 2012; Chevalier *et al.*, 2014), and MAX2/D3/RMS4 that is part of a Skp-Cullin-F-box (SCF) E3 ligase (Stirnberg *et al.*, 2002; Ishikawa *et al.*, 2005; Johnson *et al.*, 2006). The current understanding is that strigolactones are hydrolysed by D14/DAD2, upon which binding to SCF^MAX2^ is enhanced to trigger ubiquitination and target degradation of downstream signalling components, such as D53 and SLENDER1 in rice, and BRI1-EMS-SUPPRESSOR1 in *Arabidopsis* (Hamiaux *et al.*, 2012; Jiang *et al.*, 2013; Nakamura *et al.*, 2013; Wang *et al.*, 2013; Zhou *et al.*, 2013). Downstream of this early signalling complex, the TEOSINTE BRANCHED1/CYCLOIDEA/PCF1 transcription factor BRANCHED1 (BRC1) might function as an integrator of different hormonal signals to control branching (Aguilar-Martínez *et al.*, 2007; Braun *et al.*, 2012; Dun *et al.*, 2012).

Here, the importance of strigolactones in pathogen-induced changes in plant development is evaluated in leafy gall syndrome, a pathology with a strong link to apical dominance and bud outgrowth (de O Manes *et al.*, 2004; Simón-Mateo *et al.*, 2006). First, the phenotype induced by *R. fascians* strain D188 on wild-type *Arabidopsis* plants was compared with that provoked on the four *max* and the *brc1* mutants. Then, in a pharmacological approach, the importance of the endogenous strigolactone levels on symptom development was assessed by adding the synthetic racemic strigolactone mixture GR24 (Besserer *et al.*, 2006; Scaffidi *et al.*, 2014) or the CCD inhibitor D2 (Sergeant *et al.*, 2009). The expression profiles were determined of the four *MAX* genes and of *BRC1* in infected tissues of wild-type Columbia-0 (Col-0) plants and different mutants previously shown to be impaired in symptom development. Finally, diverse approaches were taken to assess the strigolactone levels in infected tissues and the impact of the bacterial cytokinins on the observed transcriptional modulations was analysed. Based on the obtained results, the latest model on the molecular basis of leafy gall formation (Stes *et al.*, 2012) was extended.

## Materials and methods

### Plant material, sampling, and infection conditions

*Arabidopsis thaliana* (L.) Heynh., accession Col-0 was used throughout the experiments. Seeds of the *max* mutant and the *max* β-glucuronidase (GUS) lines were kindly provided by Ottoline Leyser (University of Cambridge, UK), the *brc1* mutant by Pilar Cubas (Universidad Autónoma de Madrid, Spain), the *tryptophan aminotransferase1-1* (*taa1-1*) *taa1-related2-1* (*tar2-1*) [*weak ethylene insensitive8-1* (*wei8-1*) *wei2-1*] mutant by Hélène Boisivon (VIB-Ghent University, Belgium), and the *Arabidopsis histidine kinase3 (ahk3) ahk4* mutant by Tatsuo Kakimoto (Osaka University, Japan).

The seeds were sterilized and sown on half-strength Murashige and Skoog medium in a growth chamber under a 16-h/8-h light/dark photoperiod at 21±2°C. The *R. fascians* strains used were the pathogenic strain D188, containing the linear virulence plasmid pFiD188, and its plasmid-free non-pathogenic derivative D188-5 (Desomer *et al.*, 1988). These strains were grown in liquid yeast extract broth at 28°C under gentle agitation for 2 days, then diluted 100-fold in fresh medium, and allowed to grow overnight. Prior to infection, the cultures were washed and concentrated 4-fold by resuspending the bacterial pellets in sterile distilled H_2_O. *Arabidopsis* plants were infected 14 days after germination by local application of a drop of bacterial culture to the shoot apical meristem. At different time points post infection [0, 4, 7, 14, and 24 days post infection (dpi)], shoot samples for quantitative reverse-transcription-polymerase chain reaction (qRT-PCR) analysis were collected after removal of roots and flower stalks and were snap-frozen in liquid nitrogen.

### Chemical treatments

GR24 (obtained from Binne Zwanenburg, Radboud University Nijmegen, The Netherlands) was dissolved in acetone and D2 (ChemBridge Corporation; www.chembridge.com/) in dimethylsulfoxide (DMSO). The cytokinins (OlChemIm Ltd.; www.olchemin.cz) were dissolved in DMSO and supplemented to half-strength Murashige and Skoog medium at concentrations of 1 µM each for the mix of 2-isopentenyladenine (2-iP), *trans*-zeatin (tZ), *cis*-zeatin (*c*Z), and their 2-methylthio (2MeS) derivatives, or 10 μM for *t*Z. To this medium, 14-day-old plants were transferred and sampled as described above for qRT-PCR analyses after 7 days of treatment.

### RNA isolation, cDNA synthesis, and gene expression analysis

For per sample, 100mg of shoot tissue was collected and ground in liquid nitrogen. For each experiment, three biological repeats were sampled. Extraction and reverse transcription of RNA were performed as described by Stes *et al.* (2011*a*). All qRT-PCR reactions were done under the same standardized conditions: initial denaturation at 95°C for 5min, followed by 45 cycles at 95°C for 10 s, 60°C for 10 s, and 72°C for 10 s. Analysis of the data, normalized against *ACTIN2*, was as previously reported (Stes *et al.*, 2011*a*). The primer sequences are given in Table 1.

**Table 1.** Primers used for qRT-PCR amplifications

Gene	AGI	Sense	Primer sequence	Reference
*ACT2*	At3g18780	Forward	GGCTCCTCTTAACCCAAAGGC	Simón-Mateo *et al.* (2006)
		Reverse	CACACCATCACCAGAATCCAGC	
*MAX1*	At2g26170	Forward	AGACTGAGTGGACAACTTAATGAG	This work
		Reverse	GCAGAGCCAGCAAGAAGATG	
*MAX2*	At2g42620	Forward	CTCACCTCACTATCCGTGGCAAC	This work
		Reverse	CGATTGGGAGAGAAGCGAGAAGAG	
*MAX3*	At2g44990	Forward	CCTCGTCCGTACTTGGTCTAC	This work
		Reverse	TCGTCCTCTTCTTCTCCTTCTTC	
*MAX4*	At4g32810	Forward	AGAAGGTGGAAGGTGAGAG	This work
		Reverse	TGACGAGTGTGGAGTAGC	
*BRC1*	At3g18550	Forward	CTTCAGCAGCGGCGATGAG	This work
		Reverse	TTCCTCTTGTTTCGGTCGTGTTAG	

### Preparation of ethyl acetate extracts and liquid chromatography-tandem mass spectrometry analysis

Entire mock-inoculated and D188-infected Col-0 and *max4* plants were harvested at 14 dpi and 48 dpi, pooled per treatment in Erlenmeyer flasks (between 3.05g and 7.53g), submerged in ethyl acetate, and rotated at 4°C for 2 days. After filtration, the ethyl acetate extracts were washed with 0.2M KH_2_PO_4_ to remove acidic compounds, dried over anhydrous Na_2_SO_4_, and filtered. The extracts were dried under a nitrogen flow at room temperature. The liquid chromatography-tandem mass spectrometry (LC-MS/MS) analysis was done as described (Yoneyama *et al.*, 2007).

### *Orobanche minor* seed germination assay

*Orobranche minor* seeds were kindly provided by Gerda Cnops (Institute for Agricultural and Fisheries Research, Merelbeke, Belgium). The seeds were surface sterilized with 70% (v/v) EtOH containing 0.05% (v/v) sodium dodecyl sulfate for 5min and then washed with 95% (v/v) EtOH for 5min and air-dried. For the preconditioning, the seeds were sprinkled on a filter paper humidified with 1ml of sterile H_2_O in a Petri dish (5cm), sealed with parafilm, and kept in the dark at 24°C for 7 days. Excess water was removed as much as possible. For the positive control, 1ml of 0.1 µM or 1 µM GR24 solution was added; for the negative control, 1ml of H_2_O was added. Samples of the ethyl acetate extracts corresponding to 18.5mg of plant tissue were dried, dissolved in 10 µl acetone, and diluted to 1ml with water. The sample tubes were left open in the laminar flow for 30min to allow evaporation of the acetone, whereafter the samples were added to the seeds. The Petri dishes were resealed with parafilm and incubated in the dark at 24°C. After 7 days, the germination percentage was determined. All incubations were done in triplicate.

### Statistical analysis

Because assumptions for parametric tests were not met, differences in axillary activation were analysed with the Kruskal–Wallis test. When significant (*P* < 0.05), the Mann–Whitney U test for pairwise analysis, corrected with a sequential Bonferroni correction for multiple pairwise comparisons, was used.

All qRT-PCR reactions were run in triplicate, and each experiment was repeated three times. Data were compared by paired, two-tailed Student’s *t*-tests (criterion significance *P* < 0.05 for all comparisons).

## Results

### Strigolactone-related mutants display enhanced symptoms upon *R. fascians* infection

Because of the partial resemblance between the phenotype of *Arabidopsis* plants infected with *R. fascians* and that of *max* mutants, the responsiveness of the *max1-1*, *max2-1*, *max3-9*, *max4-1*, and *brc1-2* mutants (Sorefan *et al.*, 2003; Booker *et al.*, 2004; Aguilar-Martínez *et al.*, 2007; Stirnberg *et al.*, 2007) towards *R. fascians* infection was evaluated and the phenotype of the rosette was compared to that of infected wild-type plants.

**Fig. 1.**
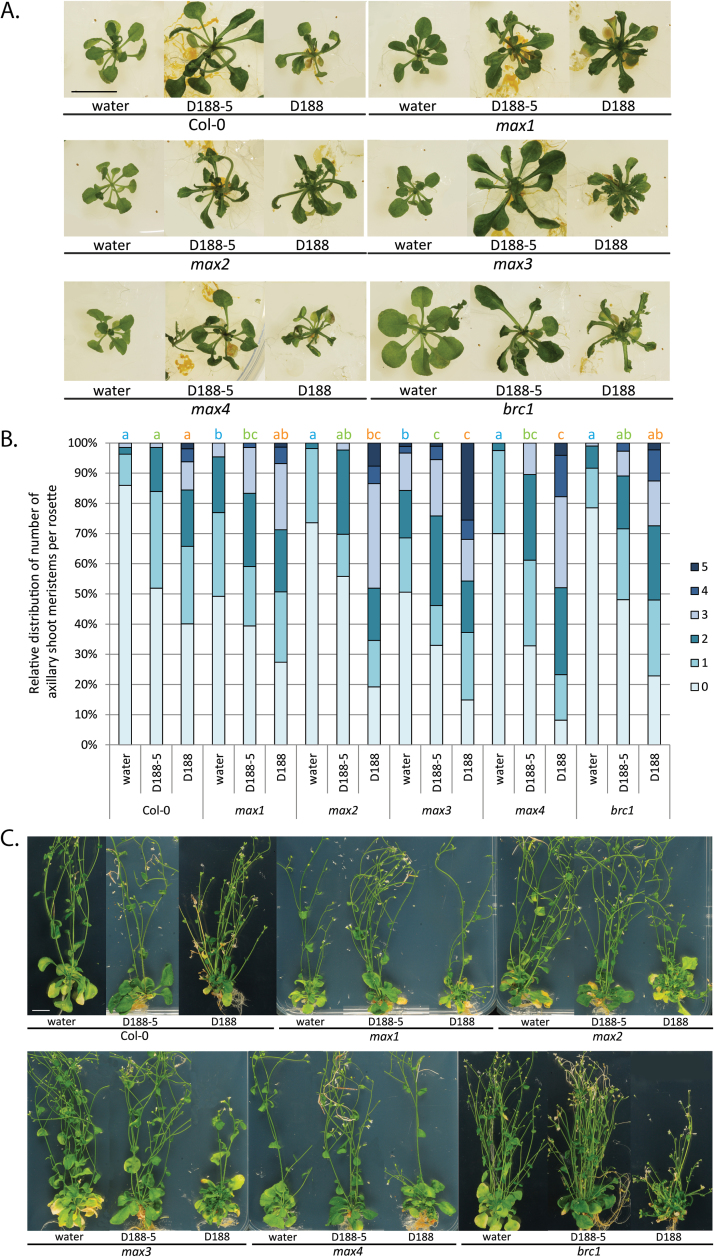
Symptom development in *Arabidopsis* Col-0 and strigolactone-related mutants upon infection with *R. fascians*. (A) Phenotype of representative plants mock-inoculated with water and infected with the non-pathogenic strain D188-5 or the virulent strain D188 at 10 dpi showing the outgrowth of shoots in axils of rosette leaves. All images were taken at the same magnification. Bar = 1cm. (B) Quantification of axillary activation (number of outgrowing shoot meristems per plant ranging between 0 and 5) at 10 dpi on at least 60 individual plants per treatment. Bars with different letters indicate a significant difference between treatments after a Kruskal–Wallis and Mann–Whitney U analysis followed by a Bonferroni correction for multiple comparisons (*P* = 0.05/n, with n = number of comparisons) (blue, water; green, D188-5; and orange, D188). (C) Phenotype of representative plants mock-inoculated with water and infected with the non-pathogenic strain D188-5 or the virulent strain D188 at 42 dpi. The effect of *R. fascians* occurs mainly in the rosette, evidenced by a bushy appearance. All images were taken at the same magnification. Bar = 1cm.

Upon infection of 2-week-old wild-type Col-0 plants with the virulent strain D188, newly developed leaves show thickened veins and serrated margins and eventually multiple axillary shoots arise from the heart of the rosette. This shoot proliferation ultimately results in bushy and often stunted plants (de O Manes *et al.*, 2004; Depuydt *et al.*, 2008; Stes *et al.*, 2011*a*; 2012) (Fig. 1A, C). Strain D188-5, a non-pathogenic plasmid-free derivative of strain D188, does not provoke leafy gall syndrome (Stes *et al.*, 2011*a*), but has a transient phytostimulatory effect on *Arabidopsis* (our unpublished data) (Fig. 1A, C). Strigolactone biosynthesis as well as the signalling mutants were all responsive towards strain D188, but, interestingly, the axillary activation in the mutants appeared to be more pronounced than that in the infected Col-0 plants (Fig. 1A), resulting in more severe bushiness of the rosettes of the mutant plants at the end of the experiment (Fig. 1C). Just as in wild-type plants, infection of the strigolactone mutants with strain D188-5 initially stimulated growth to some extent (Fig. 1A), but at the end of the experiment, no developmental changes occurred that differed from the mock-infected controls (Fig. 1C).

At 10 dpi (Fig. 1A), the axillary activation degree was scored under the binocular by counting the number of outgrowing shoot meristems in the axillary regions of the rosette leaves. For the mock-inoculated controls, only *max1* and *max3* exhibited significantly stronger loss of apical dominance than the wild-type plants at this time point (Fig. 1B) (Stirnberg *et al.*, 2002). In all plants tested, D188-5 infection induced axillary activation, possibly owing to its transient phytostimulatory effect (Fig. 1A, B). Whereas the response in the strigolactone signalling mutants was significantly higher than in wild-type plants, the axillary activation triggered by D188-5 was even higher in the three biosynthesis mutants (Fig. 1A, B). Nevertheless, of all treatments, strain D188 provoked the strongest axillary activation in all plants tested (Fig. 1A, B). The reaction of *max1* and *brc1* was comparable although more pronounced than in wild-type plants (Fig. 1A, B). The most significant differences were counted for *max2* and especially *max3* and *max4* (Fig. 1A, B), possibly suggesting that MAX2-independent pathways might contribute to the observed phenotype. Altogether, the increased developmental response upon infection of all tested strigolactone mutants compared to wild-type plants (Fig. 1A, B) illustrates their hypersensitivity towards the *R. fascians* signals.

### The efficiency of symptom development is determined by the strigolactone level

**Fig. 2.**
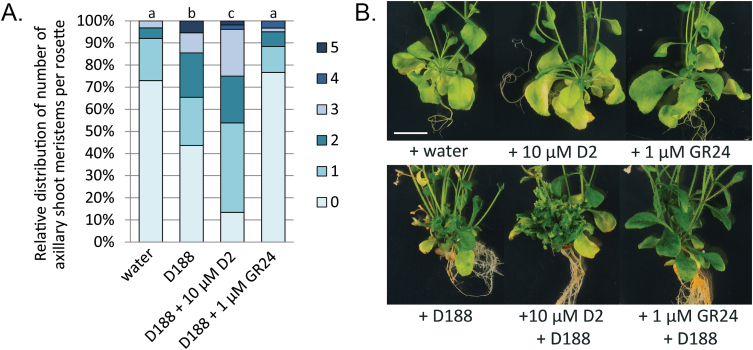
Symptom development in *Arabidopsis* Col-0 after infection with *R. fascians* strain D188 and simultaneous treatment with 10 µM D2 or1 µM GR24 showing that the strigolactone level determines the efficiency of symptom development. (A) Quantification of axillary activation (number of outgrowing shoot meristems per plant ranging between 0 and 5) at 10 dpi on at least 30 plants per treatment. Letters indicate statistical differences per treatment after a Kruskal–Wallis and Mann–Whitney U analysis followed by a Bonferroni correction for multiple comparisons (*P* = 0.05/n, with n = number of comparisons). (B) Phenotype of representative plant rosettes at 42 dpi. All images were taken at the same magnification. Bar = 1cm.

The data described above indicate that interference with stri-golactone biosynthesis and signalling has a positive impact on *R. fascians*-induced symptom development. To confirm this finding, 2-week-old wild-type Col-0 plants were transferred to media supplemented either with D2 (10 µM), an inhibitor of MAX3 and MAX4 activity, or with GR24 (1 µM), a synthetic racemic strigolactone mixture. These plants were then immediately infected with *R. fascians* strain D188. At 10 dpi, D2-inhibited strigolactone biosynthesis significantly stimulated the axillary activation triggered by strain D188 (Fig. 2A) and positively affected leafy gall formation, as evidenced by the extreme bushiness of the rosette at the end of the experiment (Fig. 2B). By contrast, GR24 reduced the axillary activation triggered by strain D188 to such an extent that the significant difference with the mock-infected control could no longer be observed (Fig. 2A). Consequently, in the presence of GR24, leafy gall formation was almost completely prevented (Fig. 2B). These results imply that the efficiency of symptom development is determined by the endogenous strigolactone levels.

### Infection with *R. fascians* transcriptionally activates the strigolactone response in *Arabidopsis*

To further investigate the involvement of strigolactones in the pathology induced by *R. fascians*, the expression profiles of the *MAX* and *BRC1* genes were determined by means of qRT-PCR on shoot tissues sampled at 0, 4, 7, 14, and 24 dpi from Col-0 plants infected with strain D188, and with strain D188-5 or mock-inoculated with water as comparative controls.

**Fig. 3.**
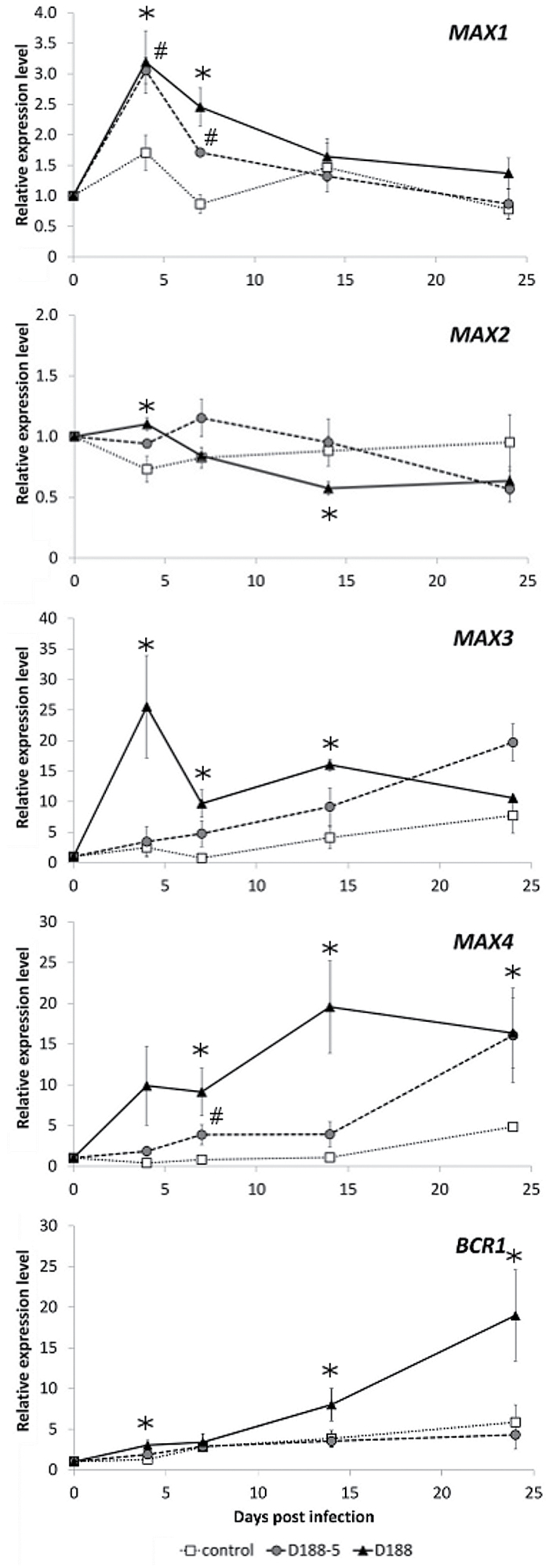
Transcript profiles obtained by qRT-PCR of strigolactone-related genes during *R. fascians*-induced symptom development in *Arabidopsis* Col-0. Error bars indicate standard errors (n = 3). Hashes and asterisks mark statistically significant differences (Student’s *t*-test; *P* < 0.05) between D188-5- and mock-infected (control) samples and between D188-infected and control samples, respectively.

In control plants, *MAX3*, *MAX4*, and *BRC1* expression displayed an upwards trend during plant development, whereas *MAX1* and *MAX2* expression did not exhibit a clear developmental regulation (Fig. 3). Infection with strain D188-5 had no significant effect on *MAX2* and *BRC1* expression, but stimulated the transcription of the three strigolactone biosynthesis genes. *MAX3* and *MAX4* transcript levels gradually increased during the interaction with strain D188-5, reaching 2- and 3-fold higher levels, respectively, than those of the mock-inoculated control at 24 dpi (Fig. 3). In contrast, *MAX1* expression was induced transiently up to 2-fold in the first week of the interaction with strain D188-5, but from 14 dpi onwards the transcript level was comparable to that of the control (Fig. 3). Infection with strain D188 provoked a similar expression profile for *MAX1*, but not for the other genes. Upon D188 infection, *MAX3* expression was transiently upregulated 5-fold at 4 dpi and subsequently decreased to a level comparable to that in control plants at 24 dpi. *MAX4* expression gradually increased during the interaction with strain D188, but this increase was faster and stronger than upon D188-5 infection until 14 dpi; at 24 dpi, infection with both *R. fascians* strains resulted in a comparable *MAX4* expression level (Fig. 3). *MAX2* expression was hardly affected by infection with D188 and did not exceed a 2-fold change (Fig. 3). From 7 days onwards, during the interaction with strain D188, *BRC1* transcription was activated to reach a 4-fold higher level than that of the controls at 24 dpi (Fig. 3). Despite the distinct expression patterns of the genes tested, the qRT-PCR analysis of host plant tissues revealed a concerted D188-triggered increase of the expression of the strigolactone biosynthesis genes at the onset of the interaction and a steady induction of the *BRC1* gene.

**Fig. 4.**
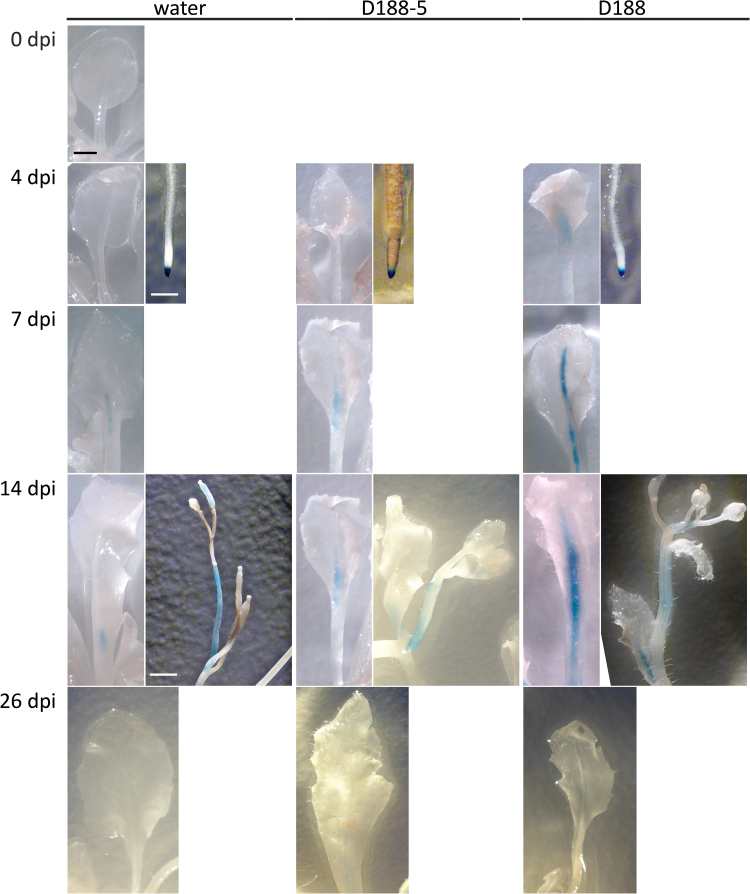
Histochemical analysis of *MAX4* expression during *R. fascians*-induced symptom development in *Arabidopsis* Col-0. Representative plant parts either mock-inoculated with water or infected with *R. fascians* strains D188-5 and D188 at different time points. At least 20 plants per time point were infected by placing a 10-µl drop of bacterial suspension at the heart of the rosette. All images for each tissue type were taken at the same magnification. Bar = 1mm.

To evaluate the effect of *R. fascians* infection on the spatial expression pattern of *MAX2* and *MAX4*, 2-week-old Col-0 plants carrying the respective promoter:*GUS* fusions (Sorefan *et al.*, 2003; Stirnberg *et al.*, 2007) were infected with strains D188-5 or D188 or mock-inoculated with water as a control; the plants were stained histochemically at 4, 7, 14, and 26 dpi. In support of the qRT-PCR data, no differences could be observed at any time point between the three different conditions for the *MAX2:GUS* line: the expression was strong in the leaves and roots, but was lower in the reproductive tissues (Supplementary Fig. S1) (Stirnberg *et al.*, 2007). By contrast, overall *MAX4* expression was weak in the mock-infected plants (Supplementary Fig. S2), except in the root tips where a strong expression was consistently detected (Fig. 4). Moreover, weak expression was occasionally observed in the floral stalks and petioles (Fig. 4), which is in agreement with the expression pattern reported by Sorefan *et al.* (2003). No ectopic *MAX4* expression could be observed upon infection with either of the *R. fascians* strains (Supplementary Fig. S2), but with D188-5 the expression in the petioles was somewhat stronger than in the mock-infected control, especially at 10 and 14 dpi (Fig. 4). Upon infection with D188, from 4 dpi onward a much stronger expression was detected in the petioles and vasculature of all symptomatic leaves (Fig. 4; Supplementary Fig. S2), supporting the qRT-PCR data. At 26 dpi, hardly any GUS staining could be observed anymore in the aerial parts of all plants tested (Fig. 4, Supplementary Fig. S2)

Thus, in contrast to what might have been predicted from the symptom development on the strigolactone mutants and the pharmacological data, the transcript data show that, upon contact with *R. fascians*, the strigolactone biosynthesis machinery is activated in the host.

### Assessing strigolactone levels in tissues infected with *R. fascians*

Although it still remains difficult to analyse strigolactones in shoot tissues of *Arabidopsis* (Seto *et al.*, 2014), the upregulation of the strigolactone biosynthesis genes upon infection with *R. fascians* was examined at the metabolite level. Shoot material was harvested at 14 dpi and 48 dpi from Col-0 plants and *max4* mutants mock-inoculated with water or infected with strain D188. The plant tissues were extracted with ethyl acetate and analysed by LC-MS/MS (see Materials and methods), but strigolactones could not be detected in any of the samples. Because seed germination of the parasitic plant *O. minor* is strongly stimulated by strigolactones (Goldwasser *et al.*, 2000, 2008), this sensitive bioassay was used to demonstrate the occurrence of strigolactones in tissues infected with D188. As a positive control, *O. minor* seeds were treated with GR24: at 0.1 µM and 1 µM, an average germination rate was obtained of 11.5% (±5.8% SE) and of 6.5% (±1.8% SE), respectively. Plant tissue extracts were prepared as for the LC-MS/MS analysis, but *O. minor* seed germination could not be stimulated by these extracts. Finally, preconditioned *O. minor* seeds were sprinkled on *Arabidopsis* roots either infected with D188 or mock-inoculated with water (48 dpi), but even after 21 days, no germination occurred. These negative results together with the increased strigolactone biosynthesis suggested by the transcription data indicate that increased strigolactone levels might occur only very localized and/or are very mild.

### Bacterial cytokinins trigger the transcriptional strigolactone response through the activation of plant auxin biosynthesis

**Fig. 5.**
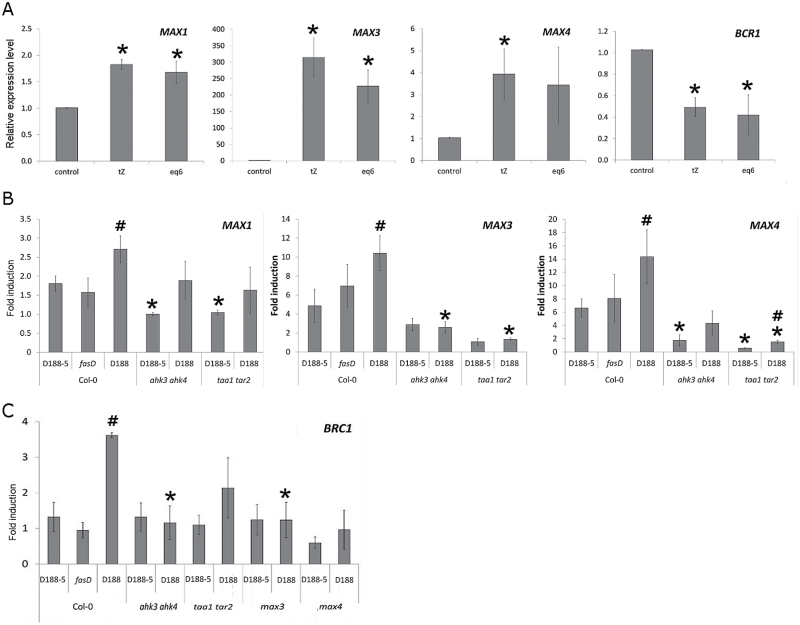
Importance of cytokinins, auxins, and strigolactones in the strigolactone transcriptional response during the interaction of *R. fascians* with *Arabidopsis*. (A) Relative expression levels of strigolactone biosynthesis and *BRC1* genes in Col-0 plants treated or not with 10 µM *t*Z or with an equimolar mix of 1 μM each of 2-iP, *t*Z, *c*Z, and their 2MeS derivatives after 7 days of treatment. Error bars are standard errors (n = 3). Asterisks mark statistically significant differences between the fold induction levels in control and cytokinin-treated plants (Student’s *t*-test; *P* < 0.05). (B) Fold induction of transcript levels of strigolactone biosynthesis genes upon infection with different *R. fascians* strains in wild-type, *ahk3 ahk4*, and *taa1 tar2* plants at 7 dpi (*R. fascians* infection versus mock infection). Error bars are standard errors (n = 3). Hashes and asterisks mark statistically significant differences between the fold induction levels obtained upon infection with the virulence-compromised *R. fascians* mutants and strain D188, and between wild-type and mutant plants, respectively (Student’s *t*-test; *P* < 0.05). (C) Fold induction of *BRC1* transcript levels upon infection with different *R. fascians* strains in wild-type, *ahk3 ahk4*, *taa1 tar2*, *max3*, and *max4* plants at 14 dpi (*R. fascians* infection versus mock infection). Statistics are as in (B).

Because cytokinins are the main pathogenicity factor of *R. fascians* and appear to be at the basis of every response analysed to date (Stes *et al.*, 2011*b*, 2012, 2013), 2-week-old Col-0 plants were placed on media supplemented either with 10 μM *t*Z or an equimolar mix of the six cytokinin bases produced by *R. fascians* (Pertry *et al.*, 2009) and shoot tissues were sampled for molecular profiling 7 days post treatment. Both cytokinin treatments induced *MAX1*, *MAX3*, and *MAX4* expression (Fig. 5A), implying that the bacterial cytokinins are important signals that can induce strigolactone-associated responses.

The impact of the *fas*-derived cytokinins on strigolactone biosynthetic gene expression was validated by determining the transcription profile of *MAX1*, *MAX3,* and *MAX4* in Col-0 upon infection with the *R. fascians* mutant D188-*fasD*, defective in the isopentenyltransferase and thus impaired in cytokinin production (Pertry *et al.*, 2010). Indeed, the expression level of the strigolactone biosynthesis genes in plants infected by D188-*fasD* was comparable to that of D188-5-infected plants and significantly lower than that measured upon D188 infection (Fig. 5B). Cytokinin perception by the plant proved to be equally important: in the double cytokinin receptor mutant *ahk3 ahk4* (Higuchi *et al.*, 2004), which is not responsive to *R. fascians* infection (Pertry *et al.*, 2009), *MAX3* and *MAX4* were no longer induced upon D188 infection, whereas *MAX1* activation was reduced (Fig. 5B). The expression level of the two *CCD* genes in the *ahk3 ahk4* mutant infected with either of the *R. fascians* strains even dropped below that measured in wild-type plants infected with strain D188-5 (Fig. 5B), indicating that expression of these strigolactone biosynthesis genes is highly sensitive, even to low levels of bacterial cytokinins.

In agreement with previous reports (Braun *et al.*, 2012; Dun *et al.*, 2012), *BRC1* expression was downregulated by exogenous cytokinins (Fig. 5A). Surprisingly, the induced *BRC1* expression seen during leafy gall development (Fig. 3) did not occur upon interaction of Col-0 plants with strain D188-*fas*D nor in the *ahk3 ahk4* mutant infected with strain D188, hinting at a cytokinin-dependent response (Fig. 5C). Based on these observations, the effect of *R. fascians* on *BRC1* expression might be the consequence of the local and/or continuous *fas*-dependent activation of strigolactone biosynthesis. Indeed, *BRC1* expression was not induced in D188-infected *max3* and *max4* mutants (Fig. 5C).

Because the *R. fascians* cytokinins target the *TAA1* and *TAR2* genes of the indole-3-pyruvic acid pathway of *Arabidopsis* that activates auxin production (Stes *et al.*, 2012) and auxin is a known inducer of *MAX3* and *MAX4* expression in *Arabidopsis* (Hayward *et al.*, 2009), the observed cytokinin-dependent increase in *MAX* gene expression upon *R. fascians* infection might possibly result from the increased auxin levels in the infected tissues. To investigate this hypothesis, the strigolactone response was analysed in the *taa1-1 tar2-1* mutant (Stepanova *et al.*, 2008). This mutant develops fewer symptomatic shoots than the wild-type plants upon D188 infection because plant-derived auxin plays an accessory role in symptom formation (Stes *et al.*, 2012). The upregulation of the strigolactone biosynthesis and *BRC1* genes observed in Col-0 plants upon D188 infection did not occur in the *taa1-1 tar2-1* mutant (Fig. 5B, C). Hence, the activation of auxin production in the host by the bacterial cytokinins seems to be responsible for the elevated transcription of the tested strigolactone genes in symptomatic tissues.

## Discussion

In the last decade, enormous progress has been made in strigolactone research. As a result, multiple roles in plant growth and development have been identified for this class of phytohormones. Moreover, based on accumulating evidence, strigolactones seem to be emerging as integrators of diverse signals involved in abiotic as well as biotic stress-related responses in plants.

Here, the impact of strigolactones was examined in a bacterial pathosystem—the interaction between *Arabidopsis* and the biotrophic actinomycete *R. fascians*—in which cytokinins are used as the main pathogenicity factor. Strigolactones were found to play a role as antagonistic compounds that restrict symptom development. Indeed, the tested strigolactone biosynthesis and sensing mutants were hypersensitive to *R. fascians* and developed stronger symptoms than the wild-type plants, with excessive development of multiple shoots from the axillary meristem regions. Moreover, symptom development was reduced in the presence of exogenous GR24 and stimulated by D2 treatment. Finally, the strigolactone biosynthesis genes were upregulated at the onset (*MAX1* and *MAX3*) and throughout (MAX4) the interaction. The hypersensitivity of the strigolactone-related mutants towards *R. fascians* infection was not entirely unexpected, because *max* and *brc1* mutants have a broken apical dominance (Stirnberg *et al.*, 2002) and an enhanced sensitivity to cytokinins (Dun *et al.*, 2012).

The expression data point out that strigolactones interfere with the molecular dialogue between *Arabidopsis* and *R. fascians*, especially at the onset of the interaction, ultimately constraining to some extent shoot induction and, hence, leafy gall formation. Clearly, activation of the strigolactone biosynthesis genes depends on the bacterial cytokinins that are produced by the main virulence locus *fas*, because their expression is no longer activated upon infection with the cytokinin-defective mutant strain D188-*fas*D. The perception of the bacterial cytokinins by the plant receptors AHK3 and AHK4 is equally important for induced strigolactone biosynthetic gene expression. Indeed, upon infection of the non-responsive *ahk3 ahk4* mutant with strain D188, *MAX3* and *MAX4* expression was comparable to that of wild-type plants upon infection with the non-pathogenic strain D188-5. Altogether, these results indicate that the bacterial cytokinins are the main effectors in the strigolactone response of this pathosystem. Nevertheless, the induced expression of *MAX3* and *MAX4* was also lost in the *taa1-1 tar2-1* mutant defective in auxin production, demonstrating that the effect of the bacterial cytokinins is indirect and mediated by host-derived auxin. Interestingly, based on these results, symptom development in the *taa1-1 tar2-1* mutant would be expected to be enhanced because of the lack of repressive strigolactones, but this is not the case. In contrast, the *taa1-1 tar2-1* mutant has been shown to be less symptomatic than the Col-0 wild type upon D188 infection (Stes *et al.*, 2012). All these data imply that plant-derived auxin plays multiple roles in symptom development: a direct positive effect (Stes *et al.*, 2012) and an indirect negative feedback via strigolactone biosynthesis. Finally, the enhanced strigolactone biosynthetic gene expression in *R. fascians*-infected tissues and subsequent activation of *BRC1* might prevent the outgrowth of the newly developing shoots (Poza-Carrión *et al.*, 2007), which is a hallmark of the leafy gall. Indeed, the *BRC1* induction observed in wild-type plants upon infection with strain D188 is lost in the *max3* and *max4* mutant backgrounds. Despite the clear strigolactone-dependent *BRC1* upregulation, strigolactones could not be detected in infected plant tissues, hinting at a very local or mild strigolactone accumulation. Thus, like in most developmental processes in which strigolactones are involved (Cheng *et al.*, 2013), the cross-talk between stri- golactones, cytokinins, and auxins also takes a central position during pathological plant development.

**Fig. 6.**
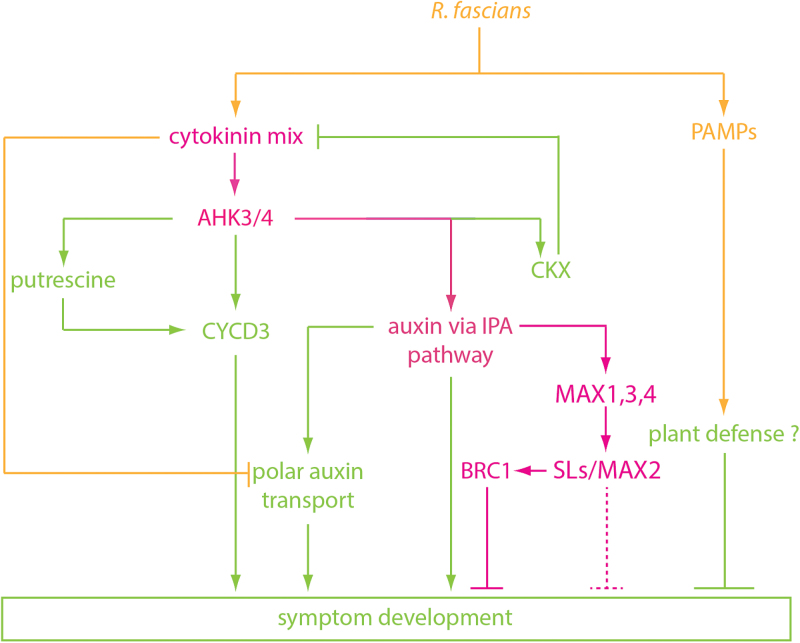
Extended model of the signalling cascade triggered by the *R. fascians* cytokinins leading to the strigolactone response (magenta) previous scheme during the *R. fascians–Arabidopsis* interaction. Orange, bacterial signals; green, plant responses. CKX, cytokinin oxidase/dehydrogenase; CYCD3, D3-type cyclin; IPA, indole pyruvic acid; PAMPs, pathogen-associated molecular patterns; SLs, strigolactones.

In conclusion, it is becoming increasingly clear that for successful leafy gall formation, balanced responses are critical. Moreover, apparently *Arabidopsis* defends itself against *R. fascians* and partially controls the impact of the bacterial cytokinins by acting on the phytohormone level. Based on the data presented, the previously proposed model (Stes *et al.*, 2012) on the molecular basis of leafy gall syndrome can be extended (Fig. 6). Upon perception of the bacterial cytokinin mix by AHK3/AHK4, the plant activates its cytokinin homeostasis mechanisms in a first attempt to counter the morphogenic pressure imposed by *R. fascians* (Depuydt *et al.*, 2008; Pertry *et al.*, 2009). The bacteria react by triggering the production of auxin and putrescine in the infected plant tissues, which function as accessory signals that aid leafy gall formation (Stes *et al.*, 2011*a*, 2012). Ultimately, in an effort to further antagonize the action of the *R. fascians* cytokinins at the target tissues, the plant locally overproduces potent inhibitors of shoot branching, the strigolactones. Through BRC1 the outgrowth of the newly induced shoots is blocked. Because BRC1 is only one of the signalling components in the strigolactone signal transduction pathway, additional direct effects of stri-golactones on symptom formation cannot be ruled out at this moment. The formation of a leafy gall unequivocally signifies that *R. fascians* wins this hormone battle. Nevertheless, although the typical defence responses generally activated in plants upon perception of pathogen-associated molecular patterns have not been addressed in detail in the *R. fascians*–*Arabidopsis* pathosystem, it would be interesting to see whether the basal immunity of the plant exerts some level of control over the pathogen to define the ideal settings for the establishment of a long-lasting biotrophic relation between *R. fascians* and its host.

## Supplementary material

Supplementary material is available at *JXB* online.

Supplementary Fig. S1. Histochemical analysis of *MAX2* expression during *R. fascians*-induced symptom development on *Arabidopsis* Col-0.

Supplementary Fig. S2. Histochemical analysis of *MAX4* expression during *R. fascians*-induced symptom development on *Arabidopsis* Col-0.

Supplementary Data

## Abbreviations:

2-iP: 2-isopentenyladenine
2MeS: 2-methylthio
AHK: *Arabidopsis* histidine kinase
BRC: BRANCHED
CCD: carotenoid cleavage dioxygenase
CKX: CYTOKININ DEHYDROGENASE/OXIDASE
*c*Z: *cis*-zeatin
D27: DWARF27
DAD: DECREASED APICAL DOMINANCE
DMSO: dimethylsulfoxide
dpi: days post infection
GR24: strigolactone analogue
GUS: β-glucuronidase
LC-MS/MS: liquid chromatography-tandem mass spectrometry
MAX: MORE AXILLARY GROWTH
qRT-PCR: quantitative reverse-transcription-polymerase chain reaction
SCF: Skp-Cullin-F-box
TAA: TRYPTOPHAN AMINOTRANSFERASE
TAR: TAA1-RELATED
*t*Z: *trans*-zeatin
WEI: WEAK ETHYLENE INSENSITIVE.
