# Supplementary material for: Strigolactones as an auxiliary hormonal defence mechanism against leafy gall syndrome in Arabidopsis thaliana
Source: J Exp Bot. 2015 Jun 30;66(16):5123–34. doi: 10.1093/jxb/erv309 (PMC4513927; doi:10.1093/jxb/erv309)
Supplement: Supplementary Data [file supp_66_16_5123__index.html]

Strigolactones as an auxiliary hormonal defence mechanism against leafy gall syndrome in Arabidopsis thaliana — Supplementary Data 

# Strigolactones as an auxiliary hormonal defence mechanism against leafy gall syndrome in *Arabidopsis thaliana*

## Supplementary Data

Data files

- Supplementary Data - Supplementary Data
