# Supplementary material for: Strigolactones as an auxiliary hormonal defence mechanism against leafy gall syndrome in Arabidopsis thaliana
Source: J Exp Bot. 2015 Jun 30;66(16):5123–34. doi: 10.1093/jxb/erv309 (PMC4513927; doi:10.1093/jxb/erv309)

Supplementary Figure S1. Histochemical analysis of *MAX2* expression during *R. fascians*-induced symptom development on *Arabidopsis* Col-0.

Representative plants either mock-inoculated with water or infected with *R. fascians* strains D188-5 and D188 at different time points. At least 20 plants were infected per time point by placing a 10-µl drop of bacterial suspension at the heart of the rosette. All images were taken at the same magnification. Bar = 1 cm.

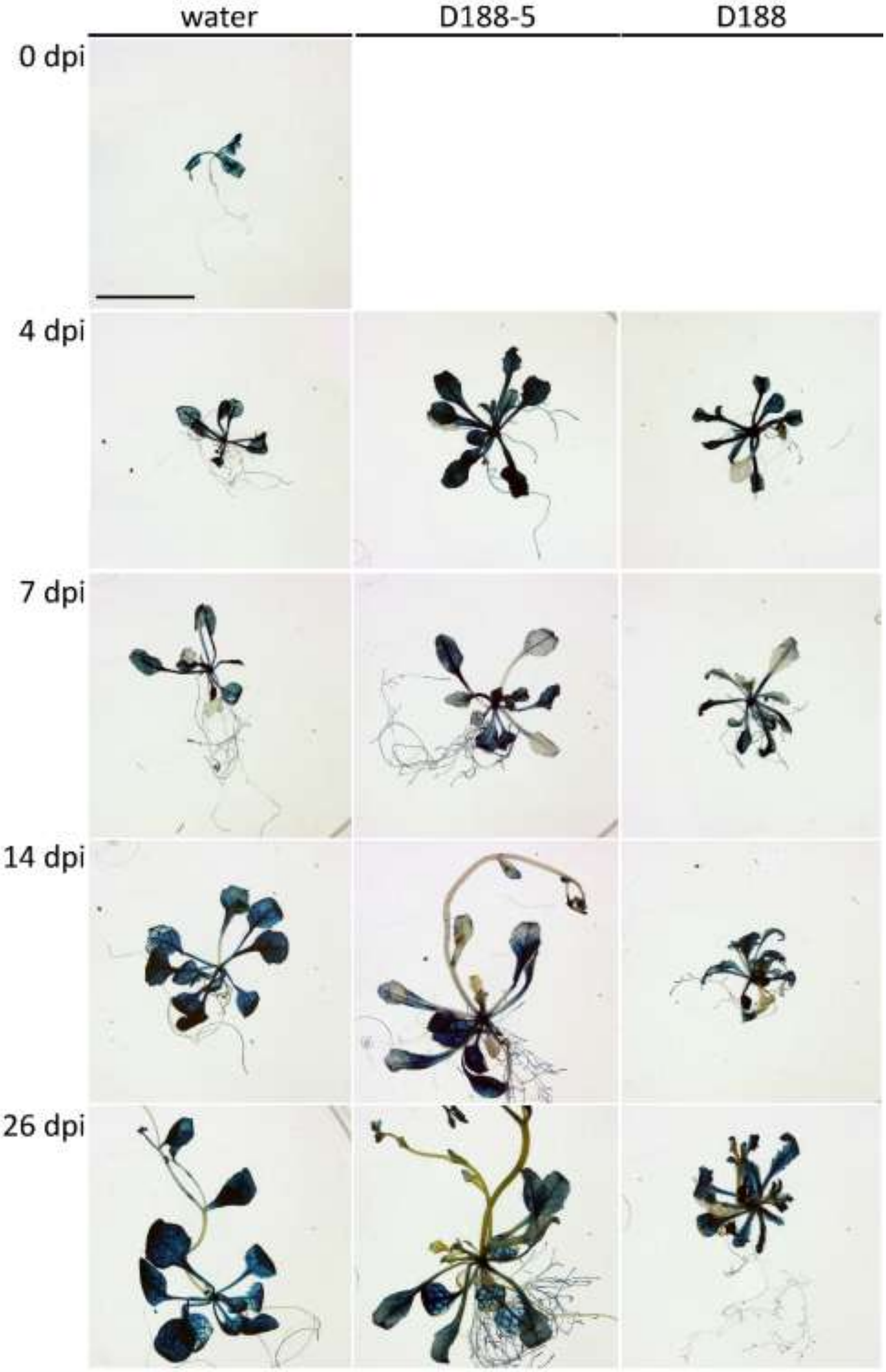

Supplementary Figure S2. Histochemical analysis of *MAX4* expression during *R. fascians*-induced symptom development on *Arabidopsis* Col-0.

Representative plants either mock-inoculated with water or infected with *R. fascians* strains D188-5 and D188 at different time points. At least 20 plants were infected per time point by placing a 10-µl drop of bacterial suspension at the heart of the rosette. All images were taken at the same magnification. Bar = 1 cm.

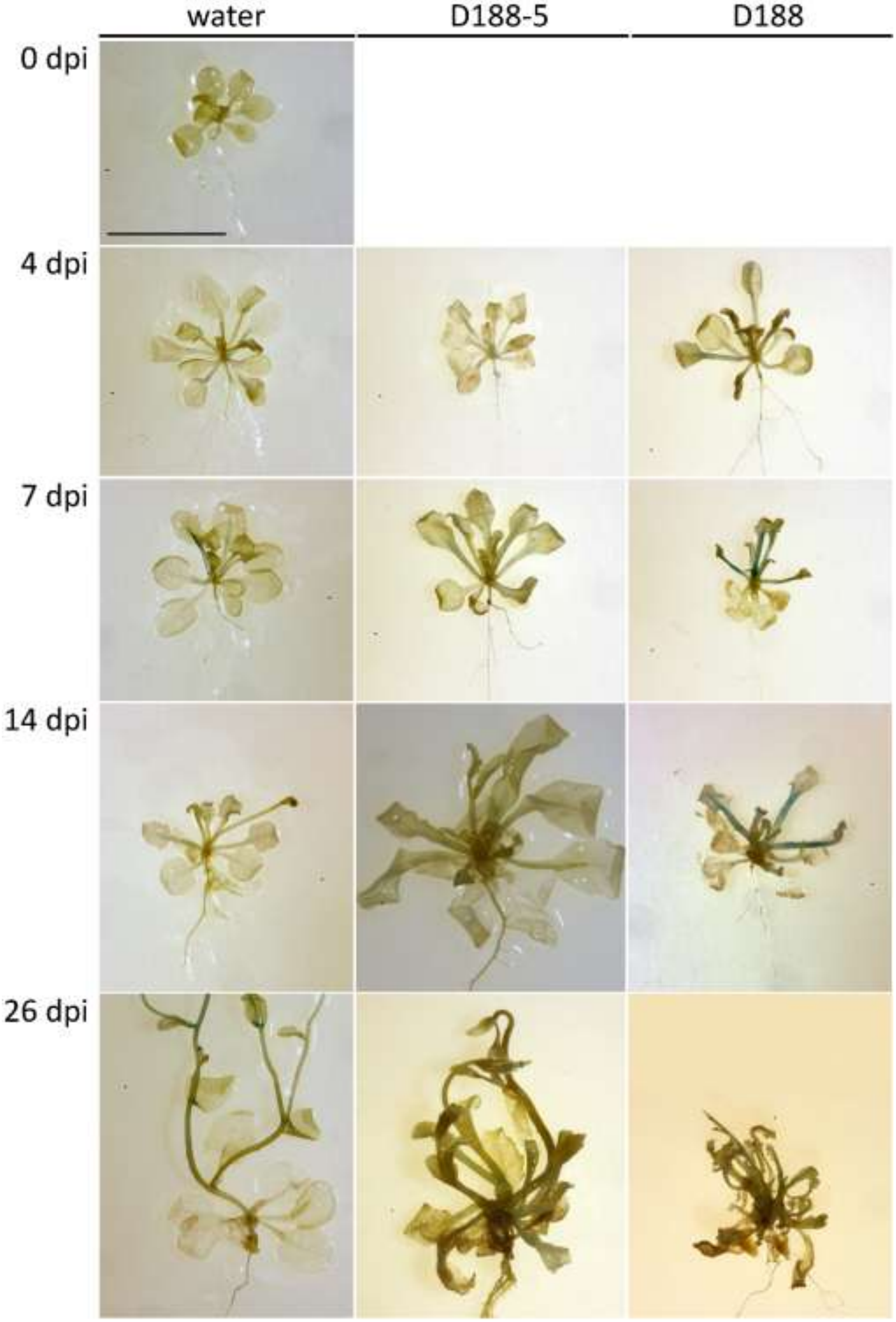

Supplement: Supplementary Data [file supp_erv309_Supplementary_material.pdf]
